# Does the targeted poverty alleviation program improve the subjective well-being of poor households? Empirical evidence from China

**DOI:** 10.3389/fpubh.2025.1717697

**Published:** 2026-01-12

**Authors:** Dazhe Wang, Xiaolei Yang

**Affiliations:** School of Economics, Nanjing University of Finance and Economics, Nanjing, China

**Keywords:** basic public services, common prosperity, difference-in-differences, subjective well-being, targeted poverty alleviation, relative poverty

## Abstract

Enhancing the subjective well-being of poor households is crucial for the world’s sustainable development. Using a comprehensive household-level dataset from the China Household Finance Survey (CHFS) spanning 2011 to 2019, this study employed a multi-period difference-in-differences (DID) approach to systematically identify the causal effect and underlying mechanisms of the Targeted Poverty Alleviation (TPA) program on the subjective well-being of poverty-stricken households. Then it explored the heterogeneous effects of different assistance measures on their subjective well-being. We found that the TPA program significantly improves the subjective well-being of rural poor households after a series of robustness checks. The analysis indicated that the TPA program improves the happiness of poor households by reducing their relative poverty and promoting their labor participation to eliminate poverty. We found that providing basic public services, means of agricultural production, and communication infrastructure all enhance the positive impact of TPA on happiness, while the housing relocation program, transportation infrastructure investment, and agricultural technical support do not. The conclusions of this study have important policy implications for ensuring equitable access to basic public services, consolidating the effective link between poverty alleviation achievements and rural revitalization in the post-poverty era, thereby promoting the common prosperity of rural households.

## 1 Introduction

Subjective well-being is the main indicator that measures people’s subjective evaluation of their quality of life (1). The 2021 World Happiness Report highlights that the bottom 1/3 of the least happy countries are predominantly developing nations, with a majority of the population engaged in agriculture (2), underscoring the importance of improving farmers’ subjective well-being. Among these specific groups, the well-being of poor farmers is often more vulnerable to a myriad of challenges, including income volatility, limited access to social security, and inadequate public services. Consequently, identifying effective pathways to enhance the rural poor’s well-being carries substantial theoretical and practical importance.

Ending poverty in all its forms everywhere in good health and well-being is ranked among the top three goals in the 17 Sustainable Development Goals (SDGs) of the United Nations 2030 Agenda for Sustainable Development. Different countries around the world have taken various measures to reduce poverty and lift people from poverty to sustainable livelihoods. As the largest developing country in the world, China’s poverty alleviation policy has made tremendous contributions to worldwide poverty reduction. Since 2013, the Chinese government has adopted a targeted poverty alleviation (TPA) program aimed at lifting around 70 million Chinese people out of poverty by 2020. From 2014 to 2018, the Chinese government concentrated antipoverty resources to improve efficiency and implement targeted measures to ensure that assistance accurately reaches poverty-stricken villages and households in accordance with the poverty alleviation standard of “Two Assurances and Three Guarantees” (liang buchou san baozhang). China also implemented “five batches” based on the causes of poverty and the needs of the impoverished people, focusing on production development, relocation, ecological compensation, education, and social security. Theoretically, the TPA program provides the rural poor with opportunities and empowerment, enabling them to have the production conditions and direction for their labor to lift themselves out of poverty, rather than simply “giving blood transfusions” to alleviate poverty. Therefore, the question worth studying is how TPA affects the subjective well-being of the rural poor and the mechanism underlying this effect.

In recent years, there has been numerous studies focus on the factors that influence subjective well-being or happiness, including income and income inequality (3–8), consumption (46), social capital (9–12), employment and entrepreneurship (13–16), housing or housing quality (17–19), environmental pollution (20), social insurance or social security (21, 47) and so on. Empirical research on the effect of the TPA program on subjective well-being is still relatively insufficient.

Several studies have focused on the economic effects of the TPA program. Lu et al. (12) found that the TPA program significantly improves the labor income and labor productivity of poor households, with relocation and industrial policies being the main channels for increasing labor supply. Li et al. (22) estimated that the TPA program has resulted in a 10.9% increase in rural income. This effect mainly arises via industrial support, agricultural development, and public service improvement. Tang et al. (23) found that the TPA program significantly reduced the urban–rural income gap, and the effect was larger in the underdeveloped western region. According to Huang and You (24), the TPA program increased GDP per capita in the 14 areas by over 45% from 2012 to 2019, with substantial gains observed in both the agricultural and non-agricultural sectors. Some scholars have also studied the effect of TPA on consumption and students’ performance. They found that the TPA significantly improved per capita consumption, survival consumption, and development consumption of poor households, and the effect was more pronounced in the poverty-stricken counties and deeply poor areas (25). Chang et al. (26) examined the effects of the TPA program on household welfare and found that the program’s effect on consumption expenditure is statistically insignificant. Nong et al. (27) showed that TPA improves the scholastic performance of girls and their achievement rank among peers. The most relevant literature to this study is Zhou et al. (28), who found that the TPA policy has a spillover effect of improving rural poor households’ happiness, and the increment of both material and nonmaterial welfare dimensions, as well as the building of positive attitudes, improves the rural poor’s happiness status.

This study evaluates the impact of the TPA program on the subjective well-being of poverty-stricken households using nationally representative data from the China Household Finance Survey (CHFS) from 2011 to 2019. Firstly, using the multi-period Difference-in-Differences (DID) approach, we found that the TPA program significantly improves the subjective well-being of rural poor households, and the treatment effect is increasing year by year. Secondly, this study explores the underlying mechanisms by which the TPA program affects their happiness, and results showed that the TPA program improves poor households’ happiness by reducing their relative poverty and promoting their intrinsic motivation to increase labor supply. Thirdly, we further explored the heterogeneous effects of TPA on subjective well-being among poor households with different causes of poverty and different assistance measures. We find that TPA significantly improves the subjective well-being of poor households living in poverty due to illness or a lack of labor force. Meanwhile, the results show that basic public services, means of agricultural production, and communication infrastructure all promote the happiness effect of the TPA program.

This study not only enriches the research in the related fields of poverty reduction and subjective well-being with the case of TPA in China but also is distinguished from previous literature in the following ways: First, we quantitatively estimate the welfare effect of the TPA program from the perspective of subjective well-being by using the multi-period difference in difference approach and other robustness checks. Second, most existing studies focus on the influencing channels at the subjective level, such as subjective social status, self-rated economic income level, future confidence level, etc. This study explores the mechanisms of relative poverty, consumption structure, and labor participation at the objective level. Thirdly, most existing studies have divided the TPA assistant measures into “economic support” and “ability cultivation” and explored the heterogeneity of policy effects. We classified the assistant measures into three categories, including basic public services (e.g., medical and healthcare, education assistance, housing reconstruction, relocation, employment, and entrepreneurship service), industry support (e.g., agricultural means of production and agricultural technology guidance) and infrastructure investment (communication and transportation investment), which enables more precise heterogeneity analysis and provides a clear pathway for policy optimization.

The rest of the study is organized as follows. Section 2 provides a theoretical framework; Section 3 introduces the research design, including the data, identification strategy, and variable definition, and presents the summary statistics. Section 4 reports the empirical results and carries out the robustness test. Section 5 explores the underlying mechanisms and heterogeneous effects. Section 6 is the conclusions and discussion.

## 2 Conceptual framework

We will construct the theoretical framework to clarify the nexus between TPA policy and the rural poor’s happiness.

Amartya Sen’s Capability Approach argues that poverty is not merely a lack of income but a deprivation of basic capabilities. These substantive freedoms enable a person to achieve valuable functioning (beings and doings they have reason to value). These “functional activities” include obtaining good nutrition, having good health, participating in community life, and enjoying dignity. Therefore, evaluating the effectiveness of a poverty alleviation policy hinges on whether it has expanded the capabilities of the impoverished population, that is, whether it has provided them with greater substantial freedom to pursue the life they value. Acquiring capability can enhance rural poor families’ resilience to risks, reduce uncertainty in life, weaken their strong sense of relative deprivation, and make them feel safe and happy (29, 30).

TPA policy may improve the rural poor’s subjective well-being by expanding capabilities and development opportunities. Specifically, industrial support policies, such as technical instruction or training, can help rural poor people master core production technology, which may increase their livelihood capital and thus improve their happiness level (48). Providing the rural poor with basic public services such as education, healthcare, housing relocation, entrepreneurship guidance, and employment assistance can bridge the gap in development opportunities and capabilities between them and those middle- and high-income families. Transportation infrastructure investment can increase the opportunity for rural labor to enter the external labor market and raise their wages (31), and communication infrastructure investment can narrow the “digital divide” between rural poor families and non-poor families, reduce the information acquisition cost, promote the rural economy transformation (32), expand channels for increasing farmers’ income (33), increase leisure and entertainment time, thus improving the rural poor’s happiness level.

*H1:* TPA has a direct enhancing effect on the subjective well-being of rural poor households.

The relative income theory argues that individuals do not pay much attention to their absolute income, but to their position relative to other people’s income within a specific reference group. People assess their economic situation by comparing themselves to neighbors, peers, or their past selves; this comparison generates a sense of “relative deprivation” or “relative superiority,” which is crucial to subjective well-being. Since poor families in rural China are at the bottom of the social income structure, they will suffer from a strong sense of relative deprivation, which affects the improvement of their happiness (28). TPA has significantly and specifically increased the absolute income of target families through multiple channels, such as industrial support, employment promotion, human capital accumulation, and agricultural development (22). More importantly, because the policy is systematically implemented at the community level, it has substantially improved the relative position of the entire impoverished group in the local income distribution. Several studies show that poverty alleviation policies have a positive effect on income distribution and relative income (23, 28). The positive effect of relative income on happiness and the importance of comparison effects have also been demonstrated in numerous studies (4, 5, 8, 34, 35).

Maslow’s hierarchy of needs provides a hierarchical model for fulfilling needs, from material to spiritual. This theory posits that meeting lower-level needs forms the basis for pursuing higher-level needs, and each level of fulfillment brings a corresponding sense of well-being. TPA policy may improve the rural poor’s happiness by consumption upgradation. Specifically, industrial support policy, such as providing poor families with means of agricultural production and technical instruction or training, can directly increase their agricultural income and, in turn, increase their subsistence consumption, including food and clothing. Basic public services may relax their liquidity constraints and reduce expenditures on children’s education, medical health care, and housing. Investment in transportation and communication infrastructure may increase the transportation and communication expenditures of rural poor households. These policies may increase both subsistence consumption and development consumption (25), and consumption upgradation may meet a higher level of demand among the rural poor, thereby improving the rural poor’s happiness level.

*H2a:* The TPA program may improve the rural poor’s happiness by increasing relative income or reducing relative poverty.

*H2b:* The TPA program may improve the rural poor’s happiness by upgrading the consumption structure.

Classical labor supply theory provides a third key perspective for understanding the welfare effects of TPA policies. This theory posits that individual labor supply decisions are formed under the combined influence of income and substitution effects. The income effect refers to the fact that, with wages held constant, an increase in non-labor income leads individuals to reduce labor supply and increase leisure; the substitution effect refers to the fact that rising wages increase the opportunity cost of leisure, thereby stimulating individuals to increase labor supply.

TPA policies, particularly their industrial support and employment promotion measures, coordinate these two effects by reshaping the incentive structure faced by impoverished farmers. While direct monetary assistance or unconditional cash transfers may generate an “income effect” or even foster welfare dependency (36), many transfer payments in TPA are linked to labor input or development outcomes. This “conditional” or “incentive-compatible” design aims to minimize the negative income effect and substantially increases the marginal output and expected wage rate of impoverished farmers engaged in agriculture or nearby non-agricultural labor. This enhances the “substitution effect,” increasing the opportunity cost of leisure and incentivizing farmers to work longer hours and put in more effort, thereby improving their subjective well-being.

*H3:* The TPA program may improve the rural poor’s happiness by promoting active labor participation rather than fostering passive welfare dependence.

## 3 Research design

### 3.1 Data sources

The data used in this study are from the China Household Finance Survey (CHFS) from 2011 to 2019, a nationwide survey conducted by the China Household Finance Survey and Research Center of Southwestern University of Finance and Economics. Based on the level of per capita GDP and the proportion of the non-agricultural population, the database used the PPS sampling method to collect data. The survey has been conducted every two years since 2011, and the coverage of CHFS data has gradually expanded. Among them, the 2011 data covered 25 provinces, with 12,569 individuals from 3,244 rural families actually living in the countryside. The 2013 data covered 29 provinces, with a total of 35,921 individuals from 8,932 rural families actually living in the countryside. The 2015 data covered 29 provinces and included 47,965 individuals from 11,654 rural families actually living in rural areas. The 2017 data covered 29 provinces and included 45,067 individuals from 12,732 rural families actually living in the countryside. The 2019 data covered 29 provinces and included 40,630 individuals from rural families actually living in the countryside.

The database collected detailed household demographic characteristics, micro information on assets and liabilities, income and consumption, insurance and security, etc. Different from other databases, the CHFS in 2019 asked for detailed information on the year of poverty alleviation, the specific means of poverty alleviation projects (such as medical and health care, education assistance, housing reconstruction and relocation, employment and entrepreneurship service, production material provision and technology guidance, and infrastructure investment), and the amount of various subsidies. It effectively addresses the problem of insufficient information on registered poor households in other databases and provides direct data support for the evaluation of the TPA program. After eliminating the outliers and limiting the sample to one year of data, the final total of 40,316 samples is included.

### 3.2 Identification strategy

The main research purpose of this study is to identify the causal effect of the TPA program on rural households’ subjective well-being. Since 2014, the CPC Central Committee and the State Council have formulated a series of guidelines and plans for the TPA strategy, and the Central Office and the State Office have issued the Opinions on the Solid Progress of Rural Poverty Alleviation and Development through Innovation Mechanism, which put forward six innovation mechanisms and ten key tasks. In April and June 2014, the Poverty Alleviation Office of the State Council successively issued the Work Plan and the Index System for Poverty Alleviation and Development, which required accurate identification of poor households and villages through the establishment of records. It proposed establishing electronic information archives for poor households, villages, counties, and contiguous poor areas nationwide by the end of 2014. Considering the staggered implementation process of China’s TPA policy, we exploited variations in both timing and individuals. We applied a multi-period DID strategy for identification. The model form is shown in Equation 1:


$$y_{\mathrm{it}}=\alpha_{i}+\mathrm{\beta TP}A_{\mathrm{ht}}+\text{ϕTreatmen}t_{h}\cdot t+(S\times f(t))\prime \theta +\gamma_{t}+\varepsilon_{\mathrm{it}}$$ (1)

In the above formula, the subscript i represents the respondents of each rural household; h represents the rural household; t represents the time; $y_{\mathrm{it}}$ represents the dependent variable of this study, namely the respondent i ‘s subjective well-being in the period t , the values of t are 2011, 2013, 2015, 2017, and 2019. $\mathrm{TP}A_{\mathrm{ht}}$ is the main variable of interest in this study, wherein $\mathrm{TP}A_{\mathrm{ht}}=\text{treatmen}t_{h}\times \mathrm{pos}t_{\mathrm{ht}}$ . According to the question of “Whether your family is in poverty status at present” in the CHFS questionnaire, if the respondent has been rated as a registered “poor household” (although he has been out of poverty), it is regarded as the “treatment group,” $\text{treatmen}t_{h}=1$ ; otherwise $\text{treatmen}t_{h}=0$ . $t_{h0}$ represents the time when the household h was registered as “poor household,” if, $t\geq t_{h0}$ then, $\mathrm{pos}t_{\mathrm{ht}}=1$ ; otherwise, $\mathrm{pos}t_{\mathrm{ht}}=0$ .

To satisfy the assumption of the parallel trend hypothesis, this study controls the interaction terms between the dummy variables of the treatment group and the time trend to control for the linear time trend differences between the treatment group and the control group. Referring to Li et al. (37), this study assumes that the influence of control variables on happiness follows a certain time trend, and adds the cross-term of control variables and the third-order polynomial of time into the model. $\alpha_{i}$ represents the individual fixed effect, which is used to control the individual characteristics of respondents that do not change over time. $\gamma_{t}$ represents the time fixed effect, which is used to control for other policy impacts in a given year. $\varepsilon_{\mathrm{it}}$ represents the random error term. Since the dependent variable is an ordered variable, it is usually more appropriate to choose the ordered Probit model for analysis. It is worth noting that Ferrer-i-Carbonell and Frijters (38) found that ML for the ordered Probit model and OLS of the simple linear regression model were consistent in the direction and significance of parameter estimation, and OLS was more intuitive and easier to interpret. Therefore, many studies directly use OLS to deal with ordered selection variable models such as subjective well-being. Especially when there are cross terms in the model, oprobit model will make the coefficient interpretation more complicated. This study directly used the panel fixed effect model for estimation.

### 3.3 Variable definition and descriptive statistics

The dependent variable of this study is respondents’ subjective well-being. According to Knight and Gunatilaka (35), Jiang et al. (8), and Zhou et al. (28), we used the self-assessment question “Considering all aspects of your life, do you feel happy right now?” from the CHFS questionnaire, assigning a value of 1 to 5 to measure the happiness level. This item has been widely used and validated by previous studies (15, 39). Several studies have proposed that reported SWB can be applied to studies of remembered and predicted utilities, and reported SWB is moderately stable (40). When happiness is used as the dependent variable, and there exists measurement error, if the error is unrelated to the independent variable, it mainly affects the significance of the estimation, not the consistency.

In terms of the selection of control variables, this study refers to the existing studies, controlling for individual characteristics, including the age of the respondents, the experience, the education level, the marital status, and the self-rated health status. Furthermore, considering that it is not random for households to be classified as “poor households” on the documented card, this study also controls for variables that both affect the entry into the treatment group and the subjective well-being of respondents. Considering that the main identification standard of registered poor households in China is that the per capita annual household income is lower than 2,300 yuan per person per year, it may also consider whether the family has a labor force, the proportion of the older population and children in the family, and so on. Without the introduction of the post-control variables (which means “bad control”), this study selected the per capita income of farmers, the proportion of older population, the proportion of children, the proportion of labor force in 2014, and the cross terms of these variables and the third-order polynomial of time as the control variables. Descriptive statistics of the main variables are shown in Table 1.

**Table 1:** Variable definitions and descriptive statistics.

Variables	Variable name	Definitions	Obs.	Mean	Std. dev	Min	Max
Dependent variable	Subjective well-being	Very unhappy = 1; Unhappy = 2; Average = 3; Happiness = 4; Very happy = 5	40,279	3.72	0.91	1	5
Main variable of interest	TPA	interaction terms of the treatment dummy variable with the time dummy variable	40,298	0.09	0.29	0	1
Control variables	Household labor force	Proportion of family members aged 16–60	36,548	0.60	0.32	0	1
	Proportion of the older population	The proportion of family members aged 60 and above	36,548	0.27	0.34	0	1
	Proportion of children	Proportion of family members aged under 16	36,548	0.13	0.16	0	1
	Household income	The logarithm of household income per capita	33,642	8.32	1.51	0.85	14.04
	Age	Age of respondent	40,316	55.64	13.00	2	113
	Experience	Divide the respondent’s age squared by 100	40,316	3.26	1.44	0.004	12.77
	Education level	Respondent’s years of education (years)	40,266	6.64	3.66	0	21
	Marital status	Unmarried = 1; Married or divorced = 0	40,316	0.03	0.16	0	1
	Self-rated health status	Very poor = 1; Bad = 2; Average = 3; Good = 4; Very good = 5	39,853	2.95	1.11	1	5

Table 2 uses the T-test to compare mean differences in key variables between poor and non-poor households. The results show that the subjective well-being of poor households is lower than that of non-poor households, which may explain the correlation between subjective well-being and the decision to enter the treatment group; that is, farmers with lower happiness are more likely to be rated as “poverty-stricken households.” Furthermore, poor households have a lower proportion of the labor force, a higher proportion of older population people, lower household income, lower level of education, and lower self-rated health status than non-poor households, which is consistent with the identification criteria of registered poor households in China.

**Table 2:** Balance test: T-test of differences between treatment and control groups.

Variables	Poor household			Non-poor households			Difference
	Obs	Mean	Std. err	Obs	Mean	Std. err	
Happiness	11,601	3.599	0.009	28,660	3.76	0.005	−0.165^***^
Household labor force	10,929	0.548	0.003	25,606	0.622	0.002	−0.074^***^
Proportion of the older population	10,929	0.322	0.003	25,606	0.248	0.002	0.074^***^
Proportion of children	10,929	0.129	0.001	25,606	0.129	0.001	0.001
Household income	10,096	7.849	0.015	23,544	8.523	0.010	−0.674^***^
Age	11,617	57.254	0.126	28,681	54.977	0.0750	2.205^***^
Experience	11,617	3.462	0.014	28,681	3.184	0.008	0.279^***^
Education level	11,598	5.538	0.035	28,650	7.092	0.021	−1.554^***^
Marital status	11,617	0.047	0.002	28,681	0.016	0.001	0.031^***^
Self-rated health status	11,472	2.667	0.011	28,363	3.058	0.006	−0.391^***^

***, **, and * denote statistical significance at the 1, 5, and 10% levels, respectively.

## 4 Empirical results

### 4.1 Baseline results

Table 3 shows the baseline regression results of the impact of the TPA program on happiness. Column (1) only controls the individual fixed effect and the time fixed effect. Based on column (1), column (2) controls the cross term between the dummy variables of the treatment group and the time trend, the individual characteristics of the respondents, and the family characteristics that may affect the rural household to be rated as a “poor household.” In column (3), the family characteristics of the respondents were replaced with different forms of cross terms of the family characteristics of the respondents and the time trend.

**Table 3:** Effects of targeted poverty alleviation policies on happiness.

Variable name	(1)	(2)	(3)
	Happiness	Happiness	Happiness
TPA	0.181^***^	0.101^***^	0.106^***^
	(0.021)	(0.031)	(0.0336)
Treatment*t		Yes	Yes
Individual characteristics of respondents		Yes	Yes
Respondents’ family characteristics		Yes	
Respondent family characteristics *t			Yes
Respondent family characteristics *t*t			Yes
Respondent family characteristics *t*t*t			Yes
Individual fixed effect	Yes	Yes	Yes
Year fixed effect	Yes	Yes	Yes
Sample size	40,261	39,756	33,168
R-squared	0.050	0.072	0.075

***, **, and * denote statistical significance at the 1, 5, and 10% levels, respectively.

The empirical results in column (1) show that, under the condition of controlling only the fixed effect, the TPA program can increase farmers’ happiness by 18.1% and the coefficient is significantly positive at the 1% significance level. However, the empirical results in column (2) show that the coefficient of influence of the TPA program on happiness significantly reduced. However, the coefficient is still significantly positive at the significance level of 1%. The decrease of the coefficient may indicate that the individual characteristics of the respondents, family characteristics, and the linear time trend difference between the treatment group and the control group also affect the probability of farmers entering the treatment group and their subjective well-being.

Compared with column (2), the empirical results in column (3) showed that the TPA program increased happiness by 10.6%, the coefficient decreased slightly, and the coefficient was significantly positive at the significance level of 1%, indicating that the family characteristics of respondents DID have some nonlinear relationship with time on subjective well-being. For example, the annual increase in farmers’ per capita income may improve farmers’ subjective well-being. Once income exceeds a certain inflection point (such as the poverty standard of 2,300 yuan per person per year), relative income level or other non-income dimension factors may lead to a more complex non-linear relationship between income and well-being. The above results all indicate that the TPA program has significantly improved the happiness, living standards, and quality of life of rural poor households.

### 4.2 Robustness check

#### 4.2.1 Parallel trend test

The assumption of the DID methodology is that the trend of change in the treatment group and the control group is consistent before the policy occurs. This study refers to Li et al. (37) and (49) to investigate the changing trends of the treatment group and the control group. To better control the unobservable factors or other policies changing over time at the provincial level, Equation 2 is set as follows:


$$\begin{array}{l} y_{\mathrm{it}}=\alpha_{i}+\beta_{1}\mathrm{TP}A_{h,\;\;t-4}+\beta_{2}\mathrm{TP}A_{h,\;\;t}+\beta_{3}\mathrm{TP}A_{h,\;\;t+2}+\\ \beta_{4}\mathrm{TP}A_{h,\;\;t+4}+\gamma X_{\mathrm{ht}}+\eta_{\mathrm{pt}}+\varepsilon_{\mathrm{it}} \end{array}$$ (2)

**Figure 1:**
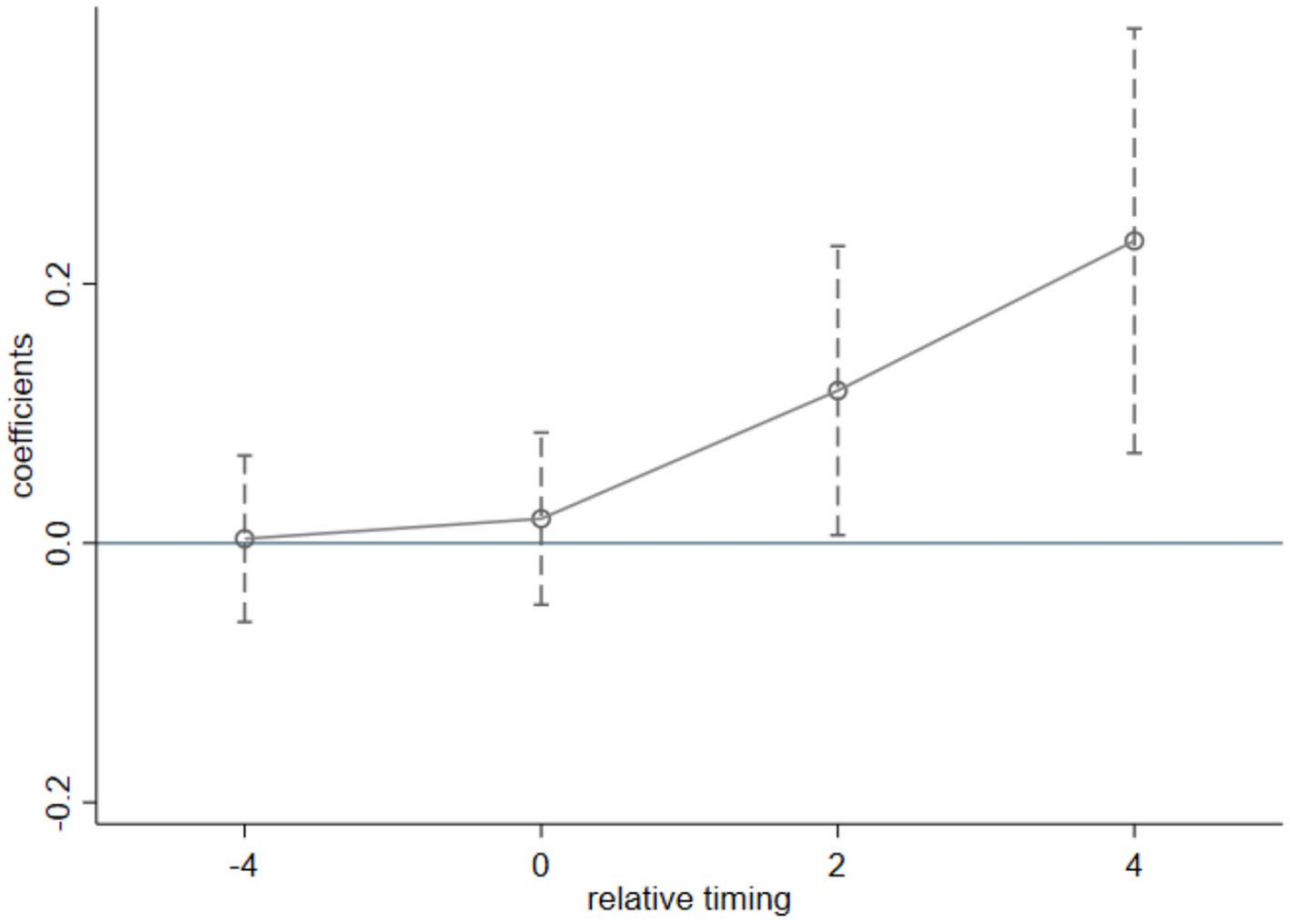
Parallel trend test.

In the above formula, TPA_h,t-4_, TPA_h,t_, TPA_h,t + 2,_ and TPA_h,t + 4_ are the dummy variables of household 4 years before, at the current time, 2 years after, and 4 years after it was rated as a poor household. u_i_ is the individual fixed effect, v_t_ is the time fixed effect, and η_pt_ is the cross-term between province dummies and time fixed effect, used to control for provincial factors that changed over time. The base group is t = −2, β_1_, β_2_, β_3_, β_4_, respectively, representing the treatment effects for the rural household rated as “registered poor household” at different times. Table 4 and Figure 1 reports the results of the parallel trend test of the TPA program on poor households’ happiness.

**Table 4:** Parallel trend test and dynamic effect.

Variable name	(1)	(2)
	Happiness	Happiness
TPA_t-4_	0.018	0.003
	(0.040)	(0.039)
TPA_t-_	0.013	0.019
	(0.041)	(0.040)
TPA_t + 2_	0.108	0.118^*^
	(0.069)	(0.068)
TPA_t + 4_	0.330^***^	0.233^***^
	(0.0536)	(0.010)
Control variables	Yes	Yes
Individual fixed effect	Yes	Yes
Year fixed effect	Yes	
Province * Year fixed effect		Yes
Sample size	33,163	33,536

***, **, and * denote statistical significance at the 1, 5, and 10% levels, respectively.

We found that the estimated coefficients of TPA_h,t-4,_ and TPA_h,t_ are not significant, which indicates that the change trends of poor households and non-poor households are the same without a significant difference before they are rated as “registered” poor households. Therefore, the model passes the parallel trend test. At the same time, we found that the estimated coefficients of TPA_h,t + 2_ and TPA_h,t + 4_ are significantly positive, which indicates that the impact of TPA policies on the well-being of poor households will increase year by year after the implement of TPA program.

#### 4.2.2 PSM-DID test

Considering that in the practice of TPA policy, the local government will comprehensively consider factors such as “Two Assurances and Three Guarantees” and whether it is easy to return to poverty in addition to taking income as the main poverty identification criteria, families with income above the poverty line are also likely to be included in the poverty system and enjoy different types of assistance means in the TPA policy. In this case, whether a household is rated as a registered poor household does not only depend on the dimension of whether its income is higher than the poverty standard, but may take into account multiple dimensions, such as the characteristics of family structure and the individual characteristics of the respondents, to determine the probability of whether it is rated as a registered poor household. In view of this, this study takes the individual characteristics of respondents and the characteristics of poor households as covariates and conducts one-to-one nearest neighbor propensity score matching. Each poor household is matched with a non-poor household whose propensity score is closest to its own. The PSM-DID method is also used to test the robustness of the regression results of the baseline model. Finally, a total of 33,069 matched samples were included. The results showed that the TPA program has significantly increased the subjective well-being of poor households by 10.5%, and this effect remains robust.

#### 4.2.3 Placebo test

**Figure 2:**
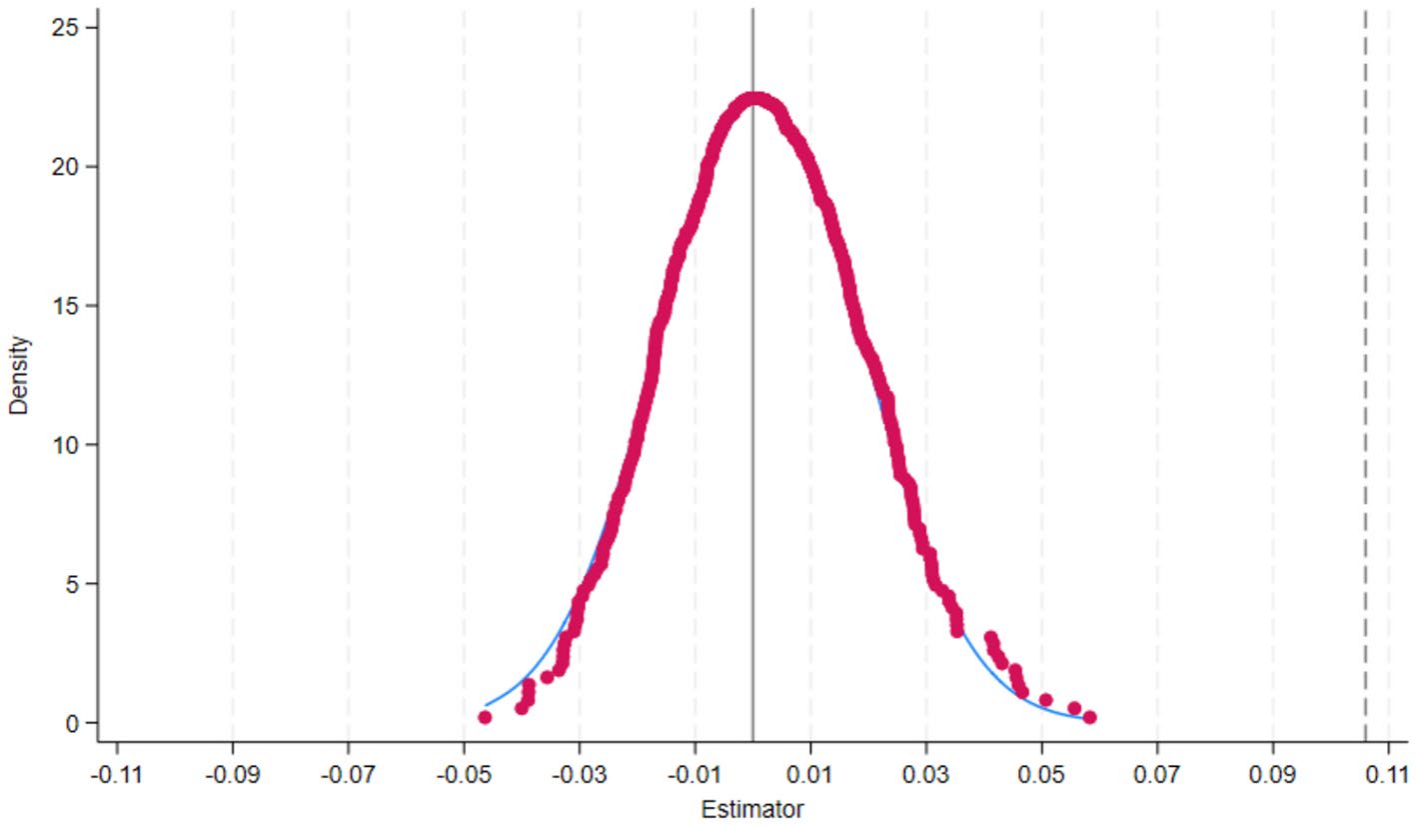
Placebo test.

**Figure 3:**
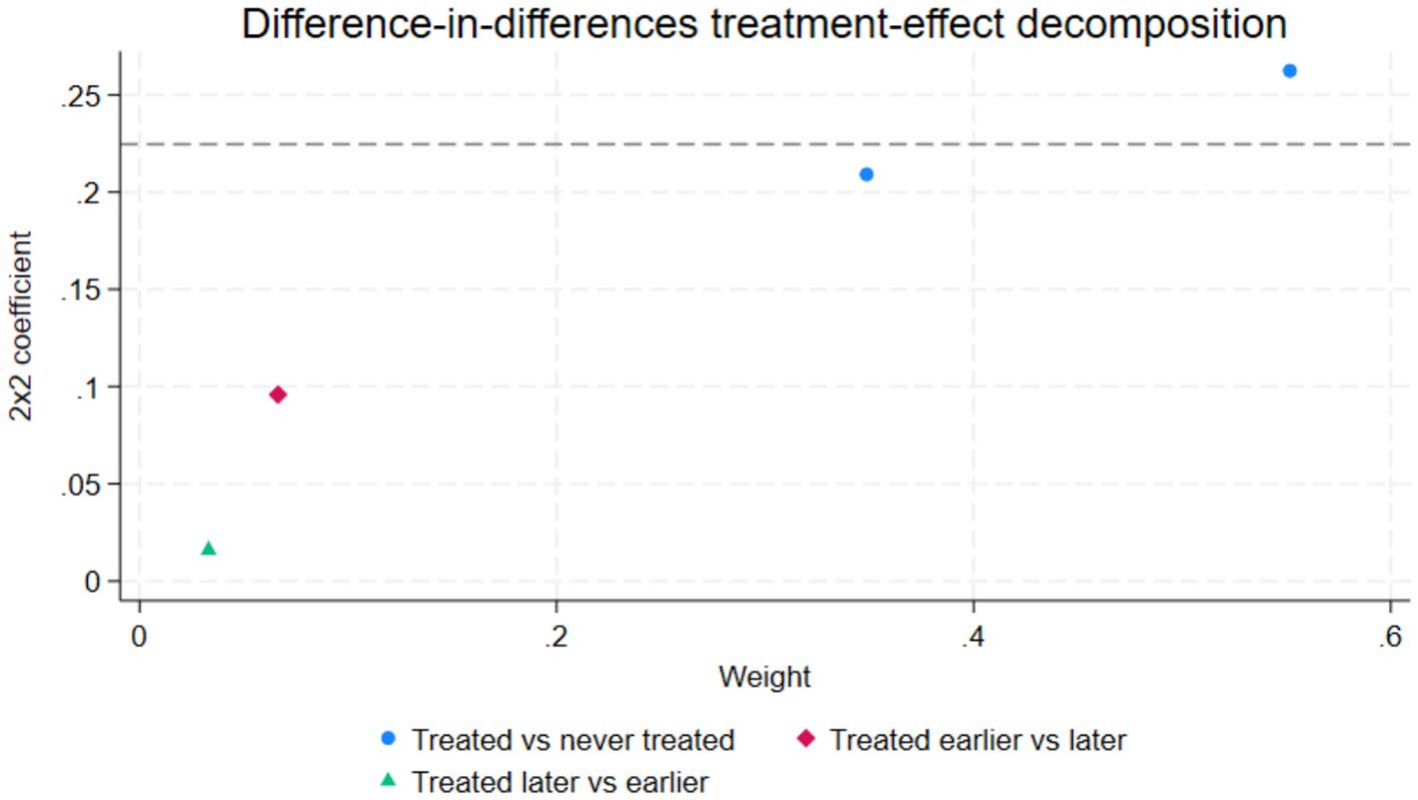
Bacon decomposition result.

Specifically, we randomly generated treatment groups from the samples of the previous period, 2014, for the policy, and then tested the effect of the policy on randomly generated treatment groups. As depicted in Figure 2, the estimated coefficient of the policy effect of the randomly generated treatment group should not significantly reject zero. The policy item TPA of the “pseudo-treatment group” should not have a significant impact on happiness, that is, TPA = 0. Otherwise, it may indicate that the DID model setting is incorrect. To improve the recognition ability of the placebo test, we simulated this process 500 times using Monte Carlo. The average estimated coefficient of the policy effect of the randomly generated treatment group was close to 0, indicating that the randomly generated “pseudo-treatment group” did not produce the policy effect. This result indicates that the promotion effect of the TPA program on happiness is not substantially biased by missing variables (Figure 3) shows the bacon decomposition result not the Placebo test.

#### 4.2.4 Test of the heterogeneity treatment effects: Bacon decomposition

Due to the different timing of the rural household was registered as “poor household” in each county, and the fact that the net effect of TPA program changes over time, the double-difference estimation method may produce errors that cannot be addressed by conventional parallel trend tests. For this reason, drawing on Goodman Bacon’s decomposition method proposed for DID, which decomposes the regression coefficients into three components: the estimated coefficients and weights of DID for the early rural household as the treatment group and the late rural household as the control group, the estimated coefficients and weights of DID for the late rural household as the treatment group and the early rural household as the control group and the estimated coefficients and weights of DID between treated group and never treated group. The late-registered rural household as the treatment group and the early-registered rural household as the control group are the real reasons for the bias in the net effect of the TPA program on rural poor’s subjective well-being, which leads to the heterogeneous treatment effects. Therefore, the severity of the above problem relies on the weight and size of the third type of DID estimator. If the average treatment effects of the third type of DID are weighted more in the estimation results, it will affect the results and interpretation of causal effects (41). The specific decomposition results are shown in Table 5 and Figure 3.

**Table 5:** Bacon decomposition results.

Bacon decomposition	2 × 2 coefficient	Weight
Treated vs. never treated	0.242	0.901
Treated earlier vs. later	0.096	0.066
Treated later vs. earlier	0.016	0.033

The results in Table 5 show that the regression bias comes from the fact that accounts for 3.3% of the weight, and the estimated coefficient is 0.016, which is the smallest. The first group has the largest weight of 90.1% and the largest estimated coefficient for DID, indicating that the impact of the TPA program on the poor household happiness is mainly due to the policy, and there is no significant bias in the estimated results.

### 4.3 Other robustness checks

While the TPA is being carried out, there may exist other policies that can also affect poor household happiness. Thus, the coefficient of DID in the baseline results may contain the effects arising from other related policy shocks. We used the cross term between rural community dummies and year fixed effect to control for other policies implemented at the community level. As column (1) of Table 6 shows, the result is robust. We also excluded samples that have been out of poverty, using the imputation estimator proposed by Borusyak et al. (42) and the stacking estimator by Cengiz et al. (43), and the results are still robust.

**Table 6:** Other robustness checks.

Variable name	(1)	(2)	(3)	(4)
	Excluding other policies	Excluding samples	Imputation estimation	Stacking estimation
TPA	0.092^***^	0.095^***^	0.197^***^	0.091^*^
	(0.035)	(0.033)	(0.032)	(0.052)
Treatment*t	Yes	Yes	Yes	Yes
Individual characteristics of respondents	Yes	Yes	Yes	Yes
Respondents’ family characteristics	Yes	Yes	Yes	Yes
Respondent family Characteristics *t	Yes	Yes	Yes	Yes
Respondent family Characteristics *t*t	Yes	Yes	Yes	Yes
Respondent Family Characteristics *t*t*t	Yes	Yes	Yes	Yes
Individual fixed effect	Yes	Yes	Yes	Yes
Year fixed effect	No	Yes	Yes	Yes
Community ×Year fixed effect	Yes	No	No	No
Sample size	32,832	32,832	14,356	21,631

***, **, and * denote statistical significance at the 1, 5, and 10% levels, respectively.

## 5 Mechanism analysis

Based on the above theoretical analysis, we will examine the mechanisms through which TPA program promote the improvement of poor household’s happiness.

Firstly, we used the 60% median of per capita income in the rural community as a threshold, and define the rural household whose per capita income lower than the threshold as “relative poverty” and rated as “1.” As column (1) of Table 7 shows, we found that the TPA program reduces the relative poverty by 3.2%; however, the coefficient is significantly positive at the 10% significance level, which may imply that the poverty reduction effect of the TPA program is limited.

**Table 7:** The mechanism analysis of TPA on happiness.

Variable name	(1)	(2)	(3)	(4)	(5)	(6)
	Relative poverty	Subsistence Consumption	Development consumption	Enjoyment consumption	Agricultural production	Non-agricultural employment
TPA	−0.032^*^	0.055^***^	−0.068^***^	−0.002	0.035^**^	−0.006
	(0.017)	(0.0361)	(0.008)	(0.001)	(0.016)	(0.006)
Treatment*t	Yes	Yes	Yes	Yes	Yes	Yes
Control variable	Yes	Yes	Yes	Yes	Yes	Yes
Individual fixed effect	Yes	Yes	Yes	Yes	Yes	Yes
Year fixed effect	Yes	Yes	Yes	Yes	Yes	Yes
Sample size	33,640	33,574	32,495	32,591	33,198	33,198
R-squared	0.120	0.114	0.177	0.119	0.078	0.035

***, **, and * denote statistical significance at the 1, 5, and 10% levels, respectively.

Secondly, columns (2) and (3) report the results, which take the consumption structure as the influencing mechanism. The results showed that TPA program significantly increase poor household subsistence consumption, but decrease their development consumption, which implied that TPA program did not promote rural household consumption upgradation. This phenomenon can be explained that the TPA program primary focuses on mitigating the most critical source of misery among the poor: profound uncertainty and precariousness. The observed rise in survival consumption is a direct manifestation of this newfound sense of security, which is the fundamental driver behind the increase in happiness. Conversely, development consumption remained stagnant because it represents a riskier form of investment that households, still exhibiting strong risk aversion, are less likely to pursue. Impoverished households lack the freedom to convert resources into “development,” such as the lack of education, health, and skills, which limits their actual ability to engage in developmental consumption.

Thirdly, we examined if TPA program can foster the “welfare dependency” of rural poor household. We use the ratio of enjoyment consumption to total consumption and “whether they are engaged in agriculture or non-agricultural employment” to measure. The column (4–6) shows that the TPA program does not increase the ratio of enjoyment consumption and increases the probability that rural poor households engage in agricultural production, but not in non-agricultural employment. From a labor economics perspective, this suggests that many impoverished households are unable to effectively utilize policy resources to pursue non-agricultural employment or invest in education due to poor health, lack of skills, limited access to information, or social exclusion. Direct policy subsidies or the provision of means of production can quickly improve their level of “possessing resources,” but transforming this into ability requires longer-term and more comprehensive support. This makes them less competitive in the labor market and makes it difficult for them to obtain stable non-agricultural jobs.

## 6 Heterogeneity analysis

This study will continue to explore the heterogeneous effect of the TPA program on the subjective well-being of poor households. We first divided TPA policies into three categories: basic public services (such as medical and health care, education assistance, housing reconstruction and relocation, employment and entrepreneurship service), industry support (such as means of agricultural production and agricultural technical guidance), and infrastructure investment. We added the interaction terms of different types of policies and core explanatory variables into the baseline model, and the results are shown in Tables 8, 9.

**Table 8:** The effects of different types of assistance measures on happiness.

Variable name	(1)	(2)	(3)	(4)	(5)	(6)
	Happiness	Happiness	Happiness	Happiness	Happiness	Happiness
TPA* Medical health care	0.217 ^***^					
	(0.059)					
TPA* Education Assistance		0.207 ^**^				
		(0.082)				
TPA* Housing reconstruction			0.174^***^			
			(0.061)			
TPA* Housing Relocation				−0.044		
				(0.121)		
TPA* Entrepreneurship					0.424^**^	
					(0.202)	
TPA* Employment						0.453^**^
						(0.176)
TPA	0.078^**^	0.094^***^	0.088^***^	0.107^***^	0.104^***^	0.102^***^
	(0.034)	(0.033)	(0.034)	(0.033)	(0.033)	(0.033)
Control variables	Yes	Yes	Yes	Yes	Yes	Yes
Individual fixed effect	Yes	Yes	Yes	Yes	Yes	Yes
Year fixed effect	Yes	Yes	Yes	Yes	Yes	Yes
Sample size	33,168	33,168	33,168	33,168	33,168	33,168
R-squared	0.075	0.075	0.075	0.075	0.075	0.075

***, **, and * denote statistical significance at the 1, 5, and 10% levels, respectively.

**Table 9:** The effects of different types of assistance measures on happiness.

Variable name	(1)	(2)	(3)	(4)
	Happiness	Happiness	Happiness	Happiness
TPA* Transportation infrastructure investment	0.075			
	(0.106)			
TPA* Communication infrastructure investment		0.300^**^		
		(0.143)		
TPA* Means of agricultural production			0.221^*^	
			(0.132)	
TPA* Technical instruction/training				0.187
				(0.131)
TPA	0.105^***^	0.102^***^	0.103^***^	0.103^***^
	(0.033)	(0.033)	(0.033)	(0.033)
Control variables	Yes	Yes	Yes	Yes
Individual fixed effect	Yes	Yes	Yes	Yes
Year fixed effect	Yes	Yes	Yes	Yes
Sample size	33,168	33,168	33,168	33,168
R-squared	0.075	0.075	0.075	0.075

***, **, and * denote statistical significance at the 1, 5, and 10% levels, respectively.

The results showed that medical and health care, educational assistance, housing reconstruction, entrepreneurship guidance, employment consultation, means of agricultural production and communication infrastructure all promoted the effect of TPA program on the subjective well-being of poor households. This may indicate that basic public services have an enabling and human capital accumulation effect, which can expand the “capability set” of impoverished households by enhancing their substantive freedoms, stimulating their endogenous motivation, and improving their quality of life. The popularization of communication infrastructure, including the Internet, also improves the happiness of poor households by providing them access to information, leisure, entertainment, and online consumption. Access to the internet directly expands informational and economic capabilities, provides low-cost access to market prices, agricultural techniques, government policies, and job opportunities, thereby reducing information asymmetry, empowering better economic decisions, and fostering their digital literacy, thus improving rural poor’ s life quality.

We also find that the housing relocation program, transportation infrastructure investment, and industrial support policies did not significantly improve the happiness effect of TPA. The possible reason is that although the government launched the relocation program to facilitate the relocation of poor households from remote, inhospitable areas to places with more work opportunities and a better life, there still exists many challenges for poor households, for example, they have some difficulties in social integration into a new community, leading to feelings of social isolation, loss of identity, and increased living costs in new settlement. Furthermore, although several studies have proved the positive income effects of transportation infrastructure investment and agricultural technology diffusion, the results above may imply that there exists the “elite capture” and the benefits of new roads or advanced agricultural technologies are rarely distributed evenly. Some scholars find that road infrastructure improvements are likely to have a greater effect on the economically advantaged than on other resident groups (44). Transport expenditures can foster inequality, with high-income groups having more opportunities than underprivileged groups to use transport resources to increase their short-term income (45). Meanwhile, wealthier farmers are better positioned to adopt expensive technologies and access new markets, while poor farmers may be left behind or even pushed into debt as they try to adopt new technologies, increasing inequality within the community.

Table 10 reports the differences of the impact of TPA program on happiness among rural households with different causes of poverty. The results show that the TPA program significantly improves the happiness of poor households due to illness and the lack of labor force, but does not significantly improve the happiness of poor households due to their children’s education. In China, due to a dualistic structure, there remains an unequal distribution of educational resources between urban and rural areas, and left-behind children in rural areas cannot access high-quality compulsory education in urban areas because of institutional barriers and restriction policies.

**Table 10:** Heterogeneous impact of TPA program on happiness: different causes of poverty.

Variable name	(1)	(2)	(3)	(4)
	Happiness	Happiness	Happiness	Happiness
TPA* Poverty due to illness	0.169^***^			
	(0.051)			
TPA* Poverty due to poor education		0.194^*^		
		(0.115)		
TPA* Poverty due to children’s education			0.061	
			(0.091)	
TPA* Poverty due to a lack of labor force				0.201^***^
				(0.066)
TPA	0.077^**^	0.101^***^	0.104^***^	0.086^**^
	(0.034)	(0.033)	(0.033)	(0.033)
Control variables	Yes	Yes	Yes	Yes
Individual fixed effect	Yes	Yes	Yes	Yes
Year fixed effect	Yes	Yes	Yes	Yes
Sample size	33,168	33,168	33,168	33,168
R-squared	0.075	0.075	0.075	0.075

***, **, and * denote statistical significance at the 1, 5, and 10% levels, respectively.

## 7 Conclusions and policy implications

Although China’s poverty alleviation strategy has made tremendous contributions to worldwide poverty reduction, it still requires serious consideration to consolidate the achievements and promote common prosperity after 2020. The adjustment of policies in the post-poverty alleviation era needs to focus on preventing large-scale rural poor households from returning to poverty, emphasizing the sustainability of policy effects, and fully considering the relationship between poverty alleviation and rural revitalization. More importantly, it is necessary to enhance the subjective well-being of poor households and improve their quality of life.

There are three main findings: First, the TPA program exerts a significant and positive causal impact on the subjective well-being of rural poor households. This highlights the program’s success in addressing the psychosocial dimensions of poverty alongside its economic indicators. Second, reducing relative poverty and enhancing the intrinsic motivation of the rural poor to escape poverty play a substantial role in promoting happiness. Third, the provision of basic public services, agricultural production resources, and communication infrastructure amplifies the positive impact of the TPA program on subjective well-being. These insights offer important guidance for designing and prioritizing poverty alleviation and rural revitalization strategies. It suggests that policies should emphasize capability-enhancing and well-being-centered interventions, which directly empower households and strengthen their social protection systems.

Our findings further emphasize the importance of inclusive public policies, and this study may have the following policy implications:

First, ensure equitable access to basic public services for low-income rural households, including preschool education, compulsory schooling, high-quality healthcare, and housing security, promoting the integration of urban and rural social security systems. Additionally, modern information and communication technologies, such as big data, cloud computing, and blockchain, should be leveraged to improve the precise identification of diverse service needs among rural households.

Second, increase industry-specific support for low-income rural households. Efforts should be made to actively facilitate their participation in local industrial development, design tailored support projects based on local conditions, and foster sustainable growth across various industries. This will help create more non-agricultural employment opportunities and strengthen long-term livelihood resilience.

Third, accelerate the development of information and communication infrastructure in rural areas to foster a more inclusive digital economy. Greater attention should be paid to empowering low-income rural groups and bridging the digital divide between households of different income levels. Providing vocational training, technical guidance, and equitable access to digital tools will help enhance human capital accumulation, improve digital literacy, and bolster the capacity of low-income households to sustain their livelihoods in the long run.

## Data Availability

The datasets presented in this study can be found in online repositories. The names of the repository/repositories and accession number(s) can be found at: https://chfser.swufe.edu.cn/datas/.
