# Correction: Does the targeted poverty alleviation program improve the subjective well-being of poor households? Empirical evidence from China

**DOI:** 10.3389/fpubh.2026.1805318

**Published:** 2026-02-18

**Authors:** Dazhe Wang, Xiaolei Yang

**Affiliations:** School of Economics, Nanjing University of Finance and Economics, Nanjing, China

**Keywords:** basic public services, common prosperity, difference-in-differences, subjective well-being, targeted poverty alleviation, relative poverty

Author Xiaolei Yang was erroneously assigned as corresponding author. The correct corresponding author and first author is Dazhe Wang.

The original version of this article has been updated.

